# Severe Anemia Caused by a Colorectal Lipoma With Central Erosions: A Case Report

**DOI:** 10.7759/cureus.85768

**Published:** 2025-06-11

**Authors:** Yusuke Yoshida, Ryohei Shoji, Yuki Matsumi, Ko Watanabe, Toshiyoshi Fujiwara

**Affiliations:** 1 Department of Gastroenterological Surgery, Okayama University Graduate School of Medicine, Dentistry, and Pharmaceutical Sciences, Okayama, JPN; 2 Department of Gastroenterological Surgery, Okayama University Hospital, Okayama, JPN; 3 Department of Pathology, Okayama University Hospital, Okayama, JPN

**Keywords:** anemia, bleeding lipoma, colorectal lipoma, laparoscopic surgery, mucosal erosion

## Abstract

Colorectal lipomas are benign tumors that are often asymptomatic and discovered incidentally. In most cases, they can be managed conservatively with observation. We report the case of a man in his 70s with a colorectal lipoma located in the cecum. An investigation into his severe anemia led to the suspicion that the cecal lipoma was the underlying cause. An ileocecal resection was performed. Erosions were observed at the center of the lipoma. Although small colorectal lipomas are generally asymptomatic and rarely cause anemia, periodic endoscopic examinations are recommended. These lesions should be considered in the differential diagnosis of lower gastrointestinal bleeding.

## Introduction

Colorectal lipomas are benign tumors that are frequently asymptomatic [1]. In many cases, they are discovered incidentally during imaging, colonoscopy, surgery, or autopsy [1]. Because they are composed predominantly of fat cells and arise from the submucosa, the overlying mucosal surface is typically smooth. When the tumor becomes large, it may compress surrounding tissues and cause bleeding [2]. However, colorectal lipomas that cause bleeding with associated mucosal erosions are extremely rare. Here, we report a case of a colorectal lipoma presenting with mucosal erosions.

## Case presentation

**Figure 1:**
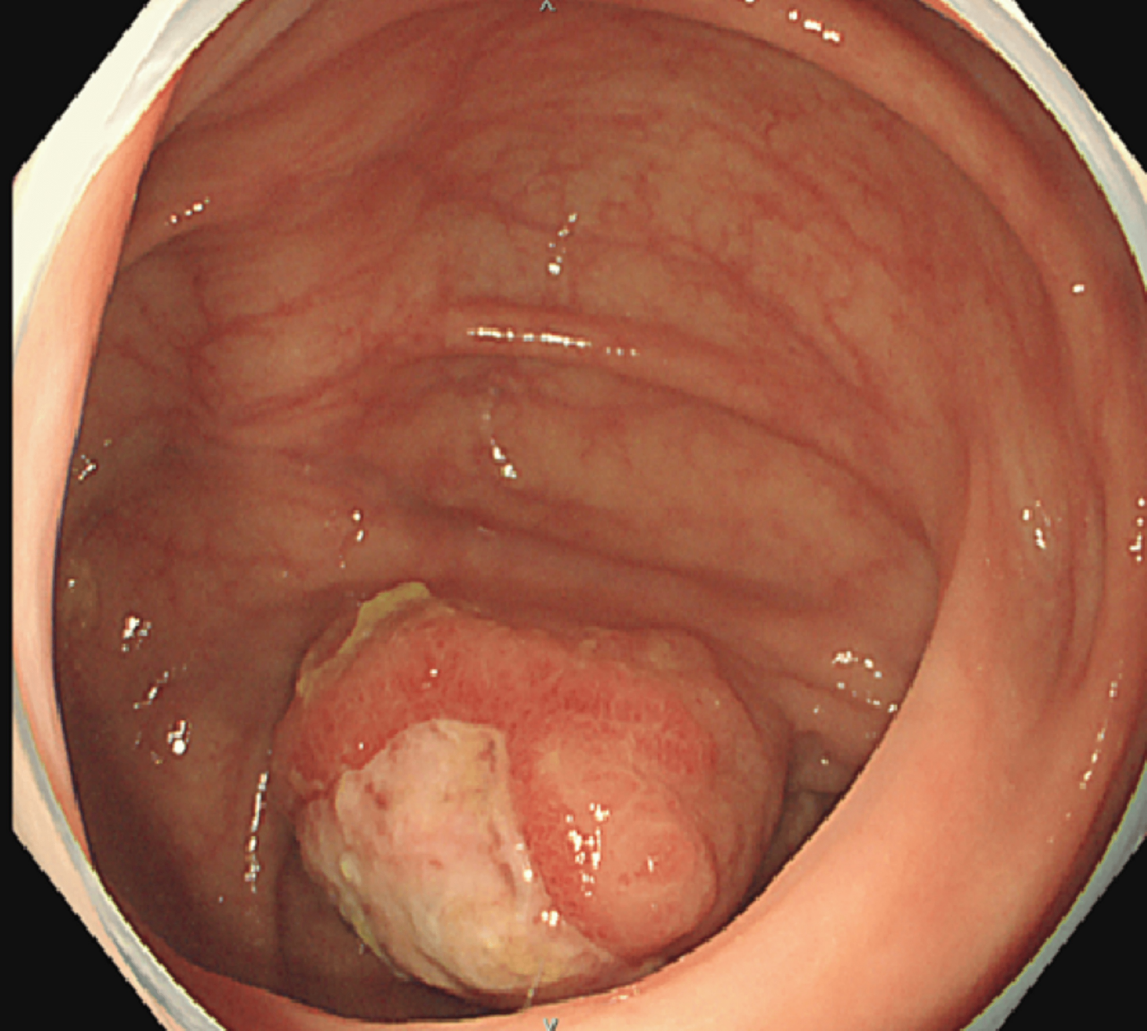
Lower gastrointestinal endoscopic image A submucosal tumor-like lesion was observed in the cecum. Ulceration with a white exudate was present at the center of the lesion, which was suspected to be the source of bleeding.

A man in his 70s undergoing maintenance hemodialysis for chronic renal failure was found to have severe anemia (hemoglobin: 6.9 g/dL; reference range: 13.7-16.8 g/dL) during a laboratory evaluation conducted due to intradialytic hypotension. Total colonoscopy revealed a 3-cm submucosal tumor with mucosal erosions located in the cecum (Figure 1).

**Figure 2:**
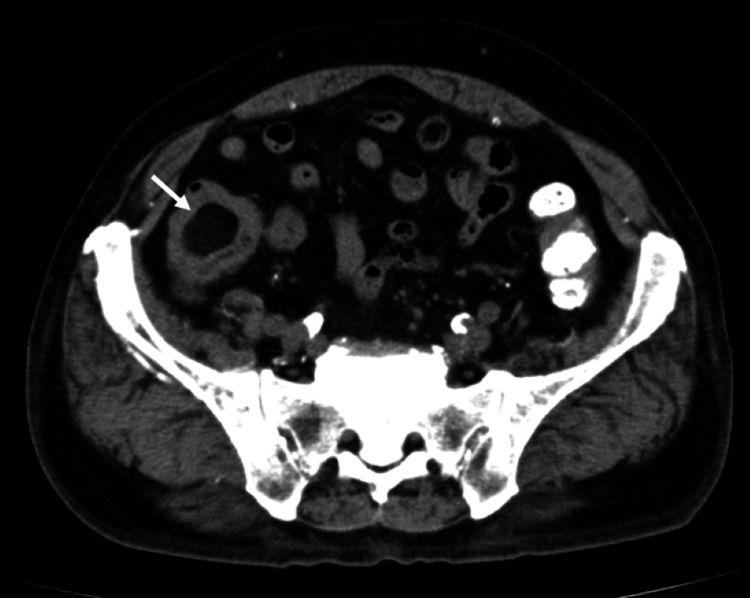
Plain abdominal computed tomography (CT) A low-density mass lesion was identified in the cecum (white arrow), suggestive of a lipoma.

Upper gastrointestinal endoscopy showed no significant findings, and the cecal lesion was suspected to be the source of gastrointestinal bleeding. Abdominal plain computed tomography (CT) revealed a low-density mass in the cecum, suggestive of a lipoma (Figure 2).

Additionally, gallstones and a common bile duct stone were detected.

Based on these findings, a laparoscopic ileocecal resection was performed under the preoperative diagnosis of cecal lipoma. Given the presence of gallstones and a common bile duct stone, the common bile duct stone was endoscopically removed prior to surgery, and a cholecystectomy was performed concurrently with the ileocecal resection.

**Figure 3:**
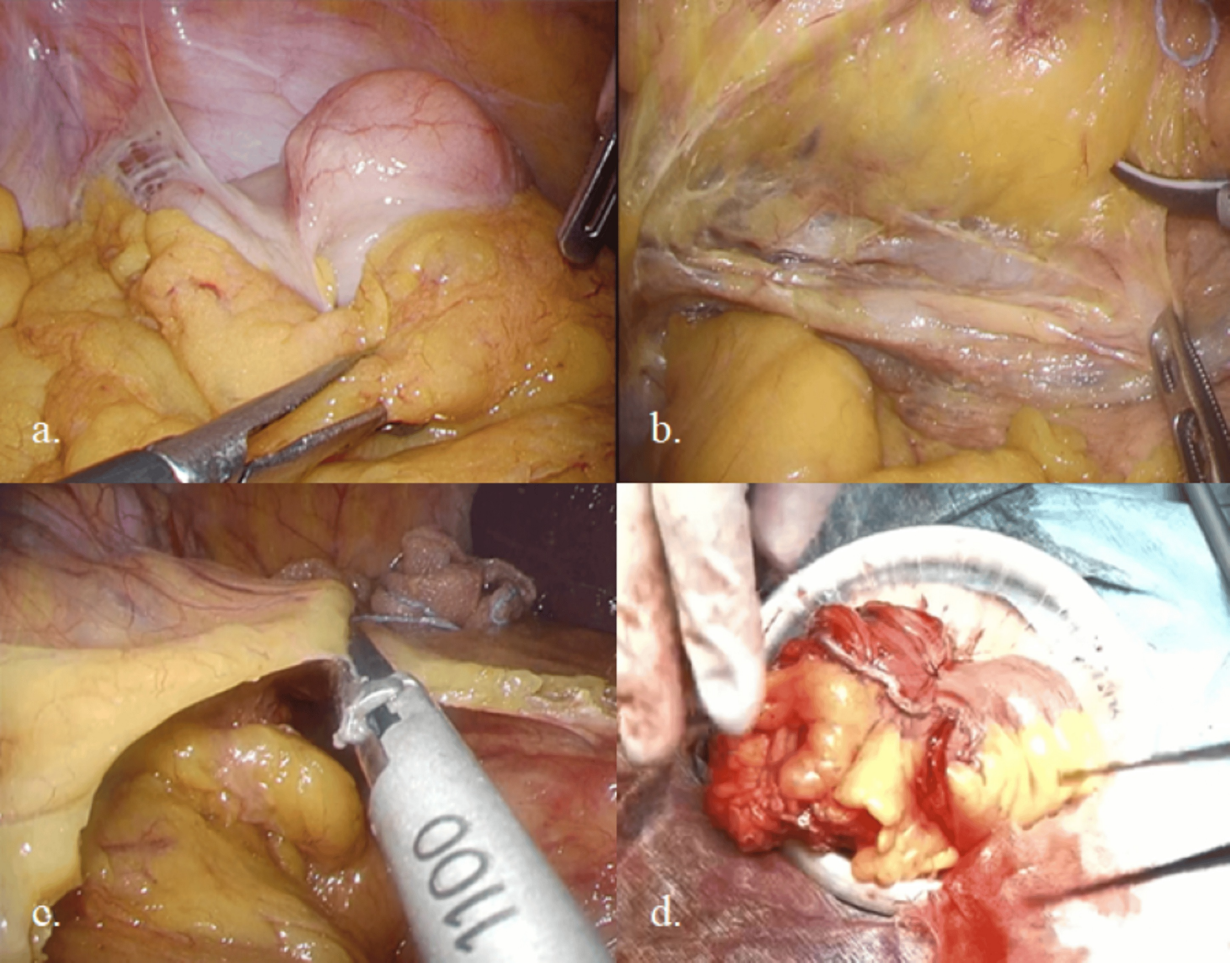
Intraoperative images of ileocecal resection (a) The cecum was fixed to the abdominal wall and was not freely mobile. (b) Ileocecal mobilization was performed by the inferior approach. (c) The hepatic flexure was mobilized from the cephalad side. (d) A functional end-to-end anastomosis was performed.

The colon surgery was initiated using the inferior approach for ileocecal mobilization, followed by mobilization of the hepatic flexure from the cephalad side (Figures 3a-3c). A cholecystectomy was then performed. Subsequently, the ileocecal resection was carried out extracorporeally using a functional end-to-end anastomosis, completing the procedure (Figure 3d).

**Figure 4:**
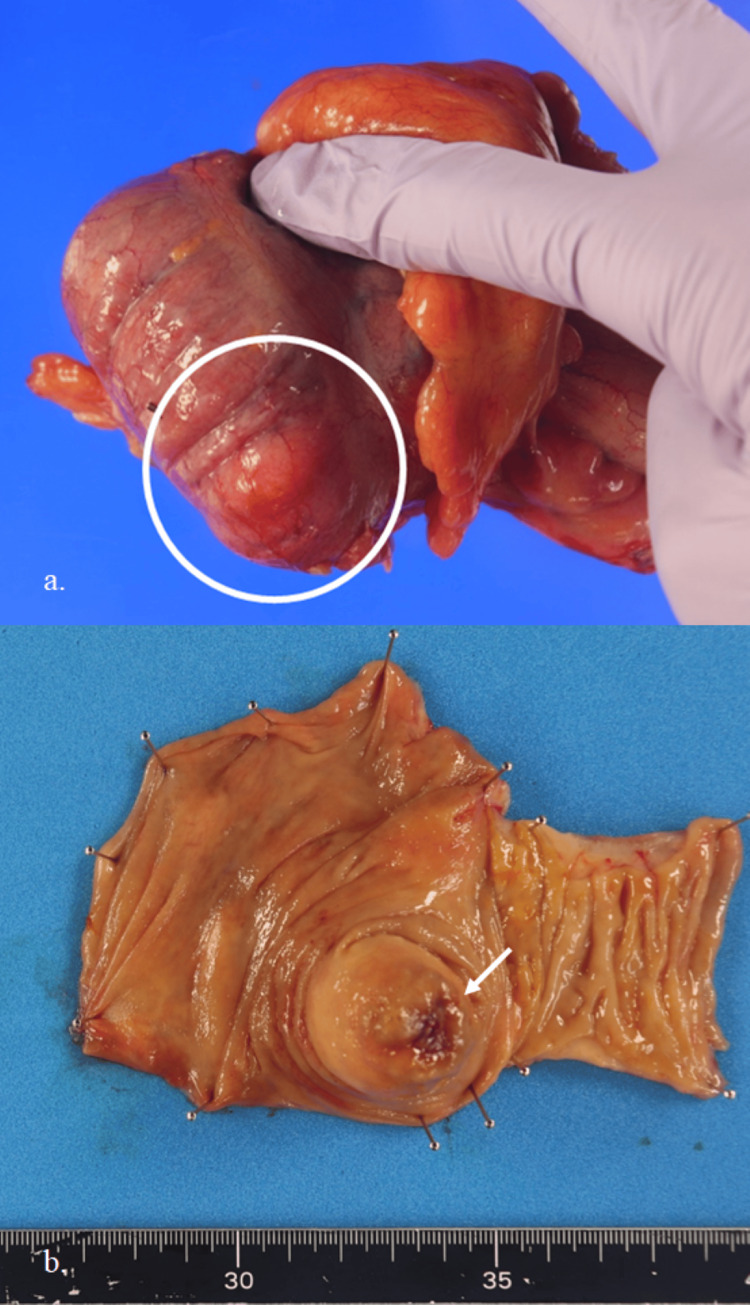
Macroscopic findings of the resected specimen (a) A yellowish submucosal tumor is seen in the cecum (white circle). (b) The center of the tumor shows mucosal erosion (white arrow).

Gross examination of the resected specimen revealed a submucosal yellow mass lesion measuring 3.1 × 1.7 cm (Figure 4a). A central mucosal erosion was noted (Figure 4b).

**Figure 5:**
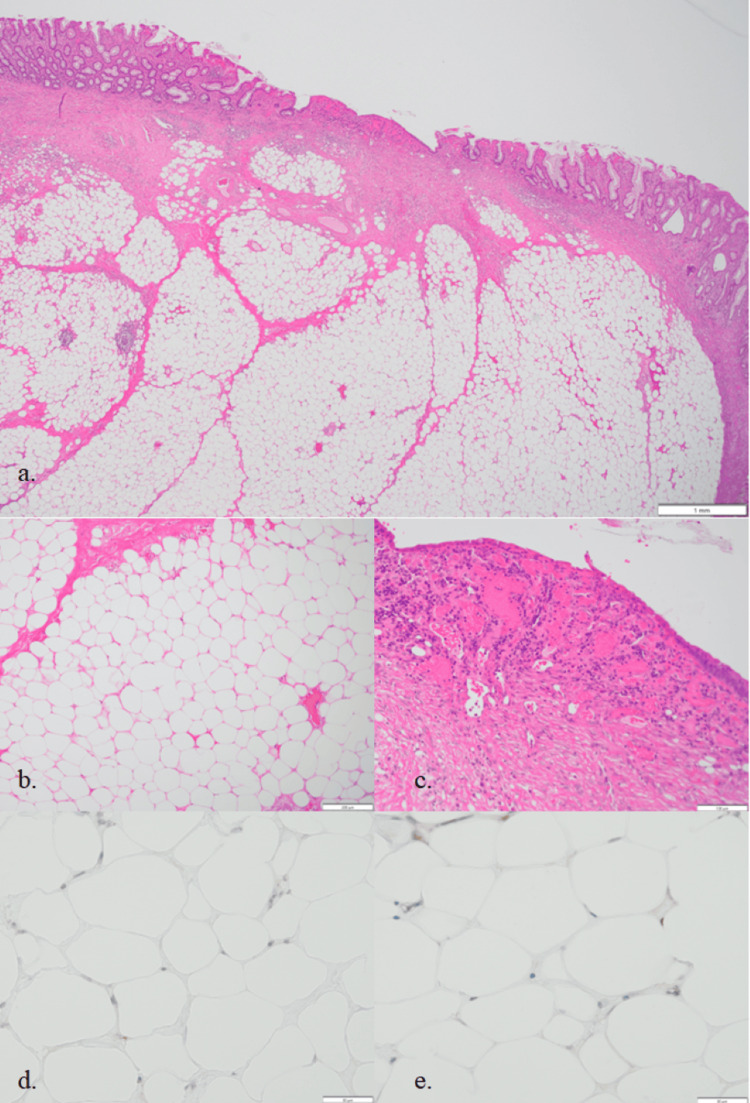
Histological findings of the resected specimen (a) Low magnification (×20), hematoxylin and eosin (H&E) staining. (b) Medium magnification (×100), H&E staining showing adipocytes characteristic of lipoma. (c) Medium magnification (×100), H&E staining showing mucosal erosion and submucosal hypervascularization. (d,e) High magnification (×400), immunohistochemical staining for MDM2 and CDK4; both were negative.

Histologically, the mass was composed of mature adipocytes interspersed with thin fibrous septa, proliferating within the submucosal layer. The septal spindle-shaped cells exhibited mild atypia; however, immunohistochemical staining for MDM2 and CDK4 was negative, confirming the diagnosis of lipoma (Figure 5). The lipoma itself was not exposed at the site of erosion. Prominent intramucosal vascular hyperplasia suggested that the erosion was caused by mechanical irritation of the mucosal surface (Figure 5c).

Postoperative bleeding was observed on postoperative day 2, with anastomotic bleeding suspected. The patient was managed conservatively and discharged on postoperative day 8.

## Discussion

In the present case, we found that a colorectal lipoma could be a potential cause of anemia.

Additionally, the lipoma exhibited central mucosal erosions.

While there are numerous reports of upper gastrointestinal bleeding caused by lipomas in the stomach, duodenum, and jejunum [3-7], reports of colorectal lipomas presenting with central erosions and associated bleeding are relatively rare, despite the colon being the most common site for gastrointestinal lipomas [6]. Hemorrhagic cases typically occur when the tumor is large [5,6]. In our case, however, the tumor was relatively small (3 cm in diameter), yet bleeding occurred.

Colorectal lipomas have frequently been reported as a cause of intestinal intussusception [8-11]. Although this patient had no typical abdominal symptoms such as pain or discomfort, we initially considered the possibility that ischemia due to intussusception had caused erosion and bleeding. However, intraoperative findings showed that the cecum was adherent to the abdominal wall (Figure 3a), and histological findings suggested that repeated intussusception was unlikely.

Therefore, we hypothesize that the bleeding was caused by mucosal desquamation due to mechanical irritation of the mucosal surface. A previous report suggested that gastrointestinal lipomas may cause bleeding due to the following: (1) impaired blood circulation in the overlying epithelium caused by tumor growth, (2) irritation by intestinal contents, and (3) mechanical irritation by peristalsis [3]. The patient’s comorbidities, including maintenance hemodialysis and the use of antithrombotic agents, may have also contributed to the severity of the hemorrhage.

The diagnosis of lipoma is typically straightforward with CT imaging, and small tumors are often asymptomatic and managed conservatively. While this is generally appropriate, our case highlights the importance of periodic endoscopic follow-up for anemia.

Another important differential diagnosis is hemangiolipoma [12], a benign tumor characterized by both adipose and vascular components. In our case, the erosive lesion consisted of intramucosal angiogenesis without tumor exposure, effectively ruling out hemangiolipoma. Although immunohistochemical staining was negative for liposarcoma markers, it remains crucial to differentiate such neoplasms, especially in tumors with rapid growth, where liposarcoma must be considered.

## Conclusions

We encountered a rare case of a small colorectal lipoma that caused severe anemia. Although lipomas can often be suspected based on CT findings and small colorectal lipomas are typically managed conservatively, periodic endoscopic examinations are essential. Additionally, because colorectal lipomas with central erosions are extremely rare, it is important to differentiate them from liposarcomas and other neoplastic lesions.
